# Psychometric adequacy of the persian adapted version of the tilburg frailty indicator (P-TFI)

**DOI:** 10.1186/s12877-024-05161-4

**Published:** 2024-07-21

**Authors:** Maryam Safarnavadeh, Leili Salehi

**Affiliations:** 1 Vice Chancellery for Education, Iran Ministry of Health and Medical Education, Tehran, Iran; 2 Department of Health Education & Promotion, School of Public Health,Research center for health,safety and environment,Karaj, Iran, Alborz University of Medical Sciences, P.O. Box 3146-883811, Karaj, Iran

**Keywords:** Tilburg frailty indicator, Validity, Reliability, Iran

## Abstract

**Background:**

Frailty is identified as the primary goal of preventing the various consequences. The present study aimed to assess validity and reliability of the Persian adapted version of the Tilburg frailty indicator (TFI) in Iran.

**Method:**

This cross-sectional study included three phases of translating the indicator to Persian, assessing the face and content validity, completing the P-TFI by older people, who helped assess the reliability and construct validity. For construct validity, convergent and divergent validity were used. It was expected that the TFI domain scores would show the highest correlations with their related measures of frailty (convergent construct validity) and the lowest correlations with measures of the other domains (divergent construct validity). The study population consisted of 400 older people, selected from six health care centers.

**Results:**

The mean age of the participants was 69.05 ± 7.28 years and the majority of the participants were married woman with less than a high school education. The total mean score of TFI was 8.26 ± 1.80, and 42.75% was classified as frail. The test-retest reliability was 0.88 for the total scale, 0.80 for physical, 0.65 for psychological, and 0.81 for social domains. The mean score of frailty and its dimensions (physical, psychological, and social) varied from 4.35 ± 1.78, 1.81 ± 1.33, 1.69 ± 0.73, and 0.86 ± 0.61, respectively. The total score of the TFI was correlated with each alternative measure and the convergent validity was proved. Further, the kappa values ranging from 535 to 0.967 were significant and test- retest reliability for total, physical, psychological, and social dimension were 0.88, 080, 065, and 081, respectively. Further, the convergent or divergent validity is being discussed for clarity.

**Conclusion:**

The Persian version of the TFI is valid and easy scored tools among Iranian’s older people.

## Introduction

Ageing is global phenomenon [1], as 20–25% of Iran’s population will be aged by the year 2031 [2]. Aging reduces various body organs’ physiological capability and functional reserve, leading to the frailty [3]. Frailty is one of the main issues experienced by the older people [4] and refers to a state of susceptibility to various side effects such as falling, inability, hospitalization, and poor quality of life [5–8]. It is estimated about 10% of the people equal and above 60 years old are frail [9]. Further, frailty is associated with decreased functional capacity and increased mortality rate [10] and identified as the primary goal of preventing the various aging consequences in many studies [11].

Considering that the frailty helps us to plan and train properly, valid and reliable tools are needed to achieve this goal. Various tools have been designed to assess the frailty, e.g., frailty index for evaluating frailty among the older people.

Frailty index was designed based on a number of health-related defects such as symptom, sign, diseases, infirmity, or laboratory measures [12] and takes a long time to complete. Groningen Frailty Indicator (GFI) focuses on disability [13], while frailty does not mean disability [14]. The fried ‘phenotype’ frailty inventory was completed through face-to-face interview and its tools were used by specialist for assessing the physical function [15]. Whereas the Tilburg Frailly Index (TFI), developed by Gobbens et al. (2010) considering the WHO health definition [14], is known as a standard self-report questionnaire with multidimensional assessment of the physical, psychological, and social dimensions, emphasizing the predictors of life expectancy, illness, and adverse outcomes such as disability, health care facilities, and death. TFI is composed of two sections, as the first section consists of 10-items and points to the frailty predictors. The second emphasizes on the disability elements and accompanies by physical, psychological, social, and inability causes by the frailty [16]. The psychometric properties of the TFI were assessed among older people in various countries [17–19]. Additionally, TFI is regarded as a valid and reproducible tool for assessing Frailty Syndrome for the Polish population [17], as well as convergent and divergent of the Brazilian scale and its items [18]. Furthermore, Italian version of the FTI has a good construct validity, since each item of the TFI is correlated with corresponding frailty measures. Convergent and divergent validity are adequate for all the domains of the TFI. Criterion validity is excellent for disability and mediocre for the fall and visiting general practitioner [19]. In the Portugal culture, TFI physical and social dimensions are correlated with concurrent measures among the older people admitted to CUs, whereas the TFI psychological domain shows similar correlations with other psychological and physical measures [20]. Furthermore, the FTI was adapted and tested among home dwelling and hospitalized older people in the Danish culture to ensure face validity and applicability of the instrument [21]. However, the desirable features of the tool have not yet been evaluated in terms of validity and reliability in Iran. Therefore, the present study aimed to translate the TFI into Persian and comprehensively evaluate its reliability and validity among a sample of community-dwelling older people in Iran. The validated tool can be used for assessing frailty among Iranian’s older people.

## Methods

### Design

**Fig. 1:**
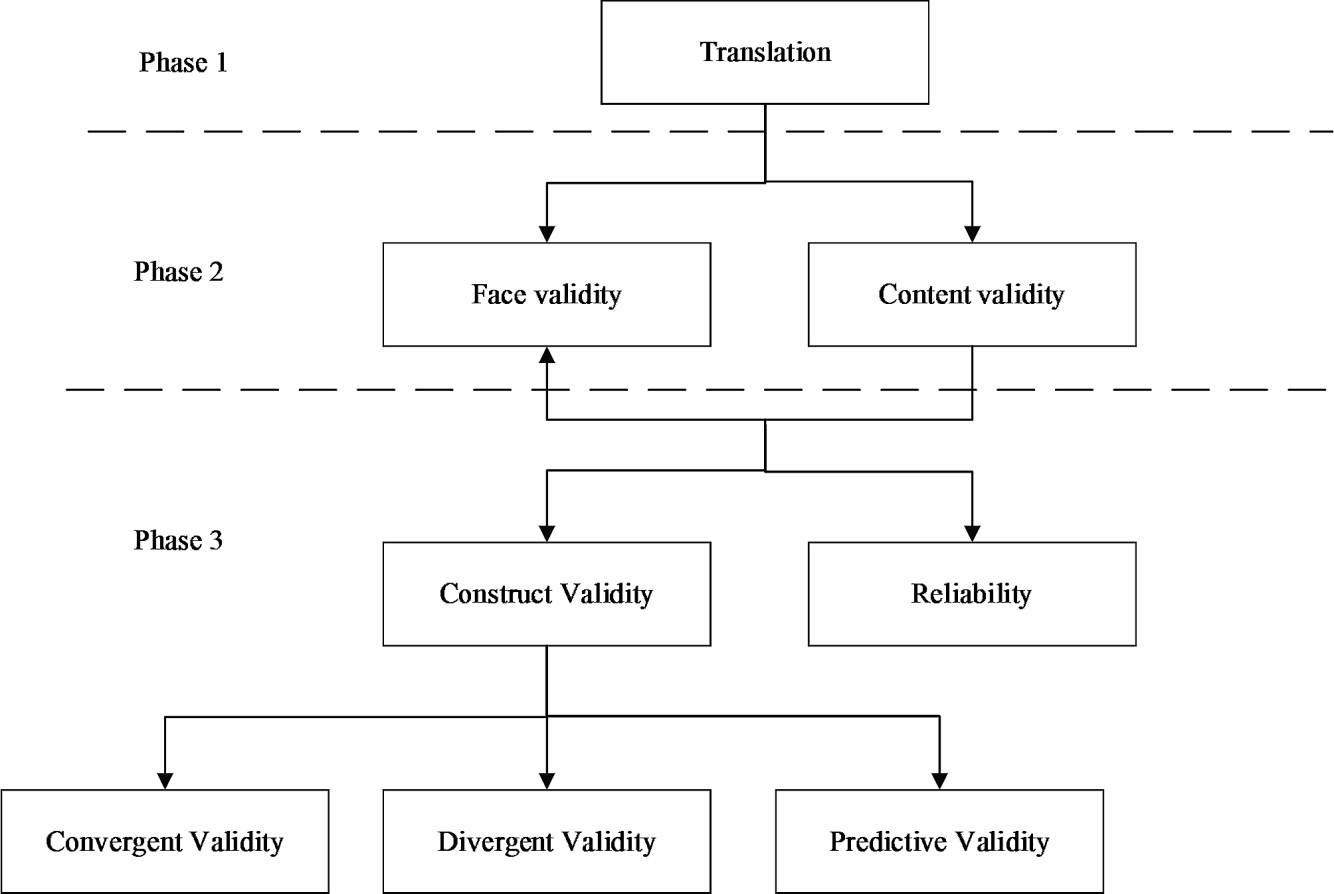
The study flowchart

This cross- sectional study included three phases and each phase composed of stages as following: [Fig. 1]

### Phase 1

#### Translation

The first version of the TFI was translated into Persian after obtaining written permission from original developer similar to the modified Brislin’s translation model [22]. According to the model, first, two top-rated English-Persian translators separately translated TFI from English into Persian, then another translator prepared the last Persian translation by combining the two main Persian translations. Afterward, two other English native translators translated the final Persian translation into English. Finally, two versions of the English back-translations and original version were compared with each other and some modifications were applied in some instances.

### Phase 2

#### Content and face validity

Ten specialists (Gerontology and public health, and health promotion) assessed TFI qualitatively and face validity was evaluated using the views of the 20 Iranian older people.

### Phase 3

During the third phase, the P-TFI was completed by older people, who helped assess reliability and construct validity.

### Reliability

As the data being dichotomous, The Kuder–Richardson substituted the Cronbach’s alpha coefficients for assessing the internal consistency. furthermore, test-retest and Cohen Kappa coefficient were used for assessing reliability. The minimum sample size was 15 subjects [23], who filled out the questionnaire two times with 2-week interval for test –retest reliability and Cohen Kappa coefficient was used for evaluating the internal consistency and 0.60 was considered as the acceptable level [24].

### Construct validity

For construct validity, convergent and divergent validity were used. It was expected that the TFI domain scores would indicate the highest correlations with their related measures of frailty (convergent construct validity) and the lowest correlations with measures of the other domains (divergent construct validity).

### Predictive validity

In addition, Activities of Daily Living and Instrumental activities of daily living were applied to examine the predictive validity. In addition, the correlation between the dimensions of TFI and the dimensions related to the quality-of-life questionnaire was assessed.

### Participations

Overall, 400 older people were included as the study population from six health care centers. According to the following formula and a systematic review related to the frailty prevalence among older people in 62 countries, sample size calculated [25].

$${\rm{n = }}\frac{{{\rm{Z}}_{\left( {{\rm{1 - \alpha /2}}} \right)}^{\rm{2}}\,{\rm{P}}\left( {{\rm{1 - P}}} \right)}}{{{{\rm{d}}^{\rm{2}}}}}$$

### P (%) = 22

Alpha = 0.05.

d (%) = 4.

For selecting the sample, six health centers were selected randomly (simple random) from a list of the Karaj health centers. In the next stage, the convenience sampling method was employed in each center to select the eligible subjects.

### Data collection

Older people received free health services from the health care centers and those aged above 60 years participated in the study and four interviewers conducted the face-to-face interview to complete the questionnaires due to a standardized procedure during 2-month. The informed written consent was obtained from all participants prior to the interview and the study followed the declaration of Helsinki.

### Measures

#### TFI- part B

The TFI included of fifteen yes/no questions about physical, psychological, and social domains of frailty and each domain contained 8-item, 4-item, and 3-item, respectively. The original developer of FTI suggests to categorize the subjects as frail, if the total score is 5 or above.

### Other measures of frailty

Various tools (Table 1) and physical indicator were applied to assess the components of frailty, including Physical frailty which is the Persian version of Physical activity for elderly (P-PASE) [26], Body Mass Index (BMI), the Gate Speed [27], TUG test [28], one item to determine poor hearing and one item to poor vision, a hand grip strength test using a changed sphygmomanometer [29], one item to assess tiredness, cognitive impairment evaluation using Mini Mental State Exam (MMSE) [30], 15-item Geriatric Depression Scale (GDS-15) for assessing feeling down [31], one item to examine nervous or anxious feeling, one item to assess coping with problems, one item to assess living arrangement, family disfunction assessment using adaptability, partnership, growth, affection, and resolve (APGAR) [32], and social support examination by the Social Support Rating Scale (SSRS) [33].

**Table 1:** Alternates measures of the TFI with continuous and dichotomous variable. Items of the TFI

Questions	Alternative measures Continuous variables	Categorical variables
1. Do you feel physically healthy?	In general, how would you rate your health? (Excellent, very good, good, fair or poor)	Excellent, very good or good = 0; fair = 1
2. Have you missing a lot of weight recently without wishing to do so?	Participants met our criteria for shrinking if they had a BMI of 18.5 kg/m2 or less, or if they reported that they had lost 5 or more kilograms in the previous year.	≥ 5% indicating shrinking (Fried et al., 2001)
3. Do you experience any difficulty in your daily life due to problematic walking?	Walking speed was measured by calculating the average time required to complete a 4-meter walk. Participants were asked to get up from a chair with a handle, walk 4 m, then turn back and walk back to the chair and sit on it. Which was measured with a stopwatch (chen, et al. 2015)	> 5 presented as difficulty in walking
4. Do you experience any difficulty in your daily life due to existence problem in maintaining your balance?	TUG test The TUG test measures the time the respondent takes to rise from an armchair, walk three meters, and return to the chair.	< 10s indicated 0 and > 10 indicated difficulty in balance
5. Do you experience any difficulty in your daily life due to poor hearing?	Making telephone calls (independent, need help or unheard)	‘Need help’ or ‘unheard’ indicating hearing impairment
6. Do you experience problems in your daily life due to poor vision?	Can you see the words on this questionnaire clearly by the distance (about 20 cm)? (Extremely, quite a bit, somewhat, a little bit or not at all)	‘Somewhat’ to ‘not at all’ indicating vision impairment
7. Do you experience any difficulties in your daily life due to lack of strength in your hands?	A modified sphygmomanometer was used to measure hand muscle strength. For this purpose, the subjects were asked to sit completely comfortably in a chair with a handle and place their hands in a comfortable position on the chair holder (at a 90-degree angle) and by holding the corrected sphygmomanometer, the strength of their hands was measured	> 135–145 mmHg considered as difficulty in hand strength
8. Do you experience any difficulties related to your physical tiredness?	How tired have you been over the past month: Full time: 1, most of the time: 2, sometimes: 3, little time: 4, never: 5 If the answer was all the time or most of the time, the score was one, and if the score was sometimes, little or never, the score was zero.	‘All of the time’ to ‘a little of the time’ indicating problems in your daily life due to physical tiredness
9. Do you have any difficulties with your retention?	The 20- items Mini Mental State Examination (MMSE)	Cognitive impairment determined by a score less than 21[ 57Cano et al., 2012]
10. Have you depressed during the last month?	The 15-item Geriatric Depression Scale (GDS-15)	≥ 4 indicating felling down
11. Have you felt furious or worry during the last month?	During the past 4 weeks, have you furious the amount of time you spent on work or other activities as a result of any emotional difficulties (such as feeling depressed or anxious) (all of the time, most of the time, some of the time, a little of the time, or none of the time)	‘All of the time’ to ‘a little of the time’ Presented dysfunctional emotion role
12. Are you able to cope with difficulties well?	I am able to do, what I want (strongly agree, agree, neutral, disagree or strongly disagree)	‘Neutral’ to ‘strongly disagree’ indicating unable to do at one’s will
13. lonely living?	who do you live with? (Living with family or living alone)	Living alone indicating positive
14. Do you sometimes miss having people around you?	The Adaptability, Partnership, Growth, Affection and Resolve (APGAR) scale	< 7 was considered as having family dysfunction
15. Do you receive adequate support from other people?	The Social Support Rating Scale (SSRS) (Xiao, 1994)	Less than the 25 were classified as having low social support

The PASE composed of 12 items related to the activities performed during the past week, and weight, frequency, and duration were evaluated for each item. The total score of the scale was obtained by multiplying the amount of time spent in each activity (h/day) or activity participation (yes/no) by the weights of the items and then, summing the results [34].

The PASE was translated and its validity and reliability were assessed by Keikavoosi-Arani and Salehi in Iran [26]. The walking speed was assessed by computing the mean time required to complete a walking until 4-meters. The participants were asked to stand up from a chair with a handle, walk 4 m, and then, go back, walk to the chair, and sit on it, which was measured with a Mobile pedometer [27] The time up and go (TUG) test measures the time takes to rise from a Morris chair, walk three meters, and return to the chair [28]. The participants with poor hearing and vision difficulties were asked to call and read questionnaire. The handgrip strength was measured by a changed sphygmomanometer used to assess strength of hand muscle. For this purpose, the subjects were asked to sit in a completely comfortable status on a chair with a catch and place their hands in a comfortable position on the chair catch (at a 90-degree angle) and the strength of their hands was assessed by keeping the corrected sphygmomanometer.

Further, boring was assessed by asking the subjects regarding their tiredness during the last 4-week and the questions were scored based on 5-point Likert scale rating from all the time to never. Mini mental status examination (MMSE) [35] with 17 items was widely used to test cognitive function such as attention, computation, memory, language, and visual-spatial skills among the older people. In addition, MMSE assessed the psychometric properties among Iranian older people and reached the optimal validation [36]. GDS is a brief scale related to the older people with depression[[37]], including 15 yes/no questions and 15 items (10 items show the depression existed among the participants and other items (1, 5, 7, 11, and 13) indicate depression in negative responses). By summing the scores of items and considering age, education, and complaints, scores 0–4, 5–8, 9–11, and 12–15 reflect normal, mild, moderate, and severe depression, respectively [38]. The validity of GDS was assessed by Malakouti et al. in Iran [39]. In addition, Activities of Daily Living (ADL) examines people’s usual daily activities and composed of feeding, bowl and bladder control, dressing and undressing, chair and bed transferring, and bathing and toileting [39] and its validity was measured in Iran [40]. Instrumental activities of daily living (IADL) indicate the participants’ difficulties with (I) ADL and consisted of seven items such as the use of telephone, shopping, food preparation, doing housework, ability to handle finances, responsibility for self-medication, and transporting out. IADL includes the activities necessary for autonomous living, including dependency, requiring partial help, and independency, scored as 0, 1, and 2, respectively. As the higher score shows the greater dependency, and IADL was validated in the Persian culture [40].

APGAR stands for Adaptability, Partnership, Growth, Affection, and Resolve (APGAR) as family function satisfaction, which consisted of 5-item, based on 3-point Likert scale (0, 1, 2) and overall ranged between 0 and 10, resulting from sum of the scores of each item [41]. Family APGAR was validated among Iranian older people by Karimi et al. (2022) [42].

Social Support Rating Scale (SSRS) [33] included 10-item and three subscales of mental social support (4-item), objective social support (3-item), and supportive behavior (3-item) and 4-point Likert scale was used to score each item and a higher score represents more social support. The old WHOQOL included 24 items and 6 dimensions (every 4 items related to a dimension) and five-points Likert scale was used to score the items. The total score of each dimension ranged from 4 to 26 [43, 44].

### Data analysis

Test-retest reliability was assessed by computing intra-class correlated coefficients (ICC) and using the Caligari Jacques categories [45]. The construct validity of the TFI was evaluated using Cohen’s kappa coefficients between each item of the TFI, and its relevant further measure, Pearson correlation coefficients between three domains of the TFI, and others frailty measures. The convergent validity was proved by statistically significant Kappa coefficients. The agreement between each item of the TFI and its related measure (dichotomized variables) was evaluated using kappa coefficients [46].

The impact score index was employed for face validity and 1.5 was considered as cut off point. Related to content validity, content validity index (CVI) and content validity ratio (CVR) were calculated[47]. Further, 0.79 was regarded as threshold for CVI and lawashe table was used for comparing obtained CVR value considering the experts numbers (in the present study, the 10 experts and 0.62 considering as threshold limit).

Additionally, the convergent validity was evaluated by statistically significant Pearson correlation coefficients. The divergent validity was expected to have higher correlations with the same domain of the TFI, and lower correlations with other domains of the TFI.

ADL disability and IADL disability were applied as outcomes to examine predictive validity of the total TFI and TFI physical domain, depression as an outcome to examine concurrent validity of the total TFI and TFI psychological domain, and low social support as an outcome to examine concurrent validity of the total TFI and TFI social domain, respectively. IBM SPSS Statistics of 19.0 was recruited to analyze the study data. In addition, one-tailed tests were used, and a *P* < 0.05 was considered as statistically significant.

## Results

### Participant characteristics

The mean age of the participants was 69.05 ± 7.28 (ranged from 60 to 93) years. The majority of the participants were female (56.8%) and other traits are presented in Table 2.

**Table 2:** The participant’s characteristics

Characteristic	*N* (%)	M ± SD
**Age (M ± SD)**		69.05 ± 7.28
60–74	313(78.25)	65.95 ± 4.17
75–84	66(16.5)	78.06 ± 2.84
> 85	22(5.5)	86.77 ± 2.28
**Sex**		
Male	173(43.3)	
Female	227(56.8)	
**Education**		
< 12	324(81.5)	
12	50(12.5)	
> 12	24(6)	
**BMI**		
< 19	9(2.25)	
19-24.9	149(37.25)	
25-29.9	169(42.25)	
> 30	73(18.25)	
**Marital Status**		
Married	286(71.5)	
Unmarried (Single, Divorce & widow)	114(28.5)	
**Co-Living**		
Alone	62(15.5)	
with spouse and child	168(42)	
With spouse without child	117(29.3)	
With child	53(13.3)	
**Economic Status**		
Appropriate	149(37.25)	
Intermediate	177(44.25)	
Inappropriate	74(18.5)	

The mean total score of TFI was 8.26 ± 1.80, and 171 participants (42.75%) were classified as frail in terms of the original cut-point of the scale (i.e., the total score ≥ 5), and considering 6 as the threshold limit for TFI (i.e., The total score ≥ 6), 89 participants (22.25) were classified as frail.

### Reliability

The Kuder-Richardson 21 (KR-21) coefficient of internal consistency reliability of the TFI was 0.81 for the total scale, 0.87 for the physical domain, 0.71 for the psychological domain, and 0.88 for the social domain. The scores for KR-21 range from 0 to 1, where 0 refers to no reliability and 1 represents perfect reliability. The closer the score to 1, the more reliable the test. In general, a score of above 0.5 is usually considered as acceptable level.

The test-retest reliability for the 14-day interval was 0.88 for the total scale, 0.80 for physical domain, 0.65 for psychological domain, and 0.81 for social domain by considering 0.6 as an acceptable level for test-retest coefficient [48].

Mean score of frailty and its dimensions varied from 4.35 ± 1.78, 1.81 ± 1.33, 1.69 ± 0.73, 0.86 ± 061, respectively (Table 3).

**Table 3:** Mean score of frailty and its dimensions (*n* = 400)

Frailty	Mean	SD	Median	Min	Max
Physical	1.81	1.33	2	0	10
Psychological	1.69	0.73	2.00	0	3
Social	0.86	0.61	1	0	1
Total	4.35	1.78	4	1	12

### Construct validity

The total score of the TFI correlated with each alternative measure as expected (Table 4). The convergent validity of the TFI was proved by the Cohen’s kappa coefficient between each item of the TFI and corresponding alternative tools. All of the kappa values ranging from 0.535 to 0.967 were statistically significant (Table 4).

**Table 4:** Construct validity (convergent validity): kappa coefficients between each item of the TFI and corresponding alternative Frailty measures

Items of TFI Physical domain	Scale	KAappa	*P*
Q11.Do you feel physically healthy?	Fair or poor health (SRH)	0.658	0.000
Q12.Have you lost a lot of weight recently without willing?	Shrinking	0.558	0.000
Q13.Do you experience problems in your daily life due to difficulty in walking?	Slowness of walking speed	0.689	0.000
Balance Q14.Do you experience problems in your daily life due to difficulty maintaining your balance?	Balance difficulty (TUGT)	. 521	0.000
Q15.Do you experience problems in your daily life due to poor hearing?	Hearing defect (making telephone call)	0.567	0.000
Q16.Do you experience problems in your daily life due to poor vision?	Vision defect (see the words on questionnaire	. 507	0.000
Q17.Do you experience problems in your daily life due to lack of strength in your hands?	Weakness (grip strength)	0.515	
Q18.Do you experience problems in your daily life due to physical tiredness?	Exhaustion (poor endurance)	0.500	0.000
Psychological domain Cognition		. 623	0.000
Q19.Do you have problems with your memory?	Cognitive impairment (MMSE)		
Mood Q20.Have you felt down during the last month?	Depression (GDS-15)	. 613 0.555	0.000
Q21.Have you felt nervous or anxious during the last month?	Dysfunctional emotion role (emotional role)	0.508	0.000
Social domain Living alone	Unable to do at one’s own will (I am able to do, what I want”	0.428 0.0.06	0.000 0.000
Q23.Do you live alone?	Living arrangement		
Social connections		0.013	0.000
Q24.Do you sometimes miss having people around you?	Family dysfunction (APGAR)		
Social support		− 0.272	0.000
*Q25.Do you receive adequate support from other people?*	*Low social support (SSRS)*		

The physical aspects were significantly correlated with the others physical measures as expected, showing a good convergent validity. Further, its divergent validity was well, since its correlations with alternative physical tools were higher than its correlations with the other frailty dimensions. Additionally, there is a significant and good correlation between it and physical QoL domain. The psychological domain had good convergent validity, due to its significant correlations with each alternative measure, while its divergent validity was not good. The social domain illustrated both good convergent and divergent validity, since the social frailty was significantly correlated with its alternative measure as expected, and correlated more strongly than did the other two domains. Furthermore, there were significant and weak to moderate correlation between psychological and social frailty and their corresponding QoL domains (Table 5). Furthermore, the study finding provided the evidence for the TFI domains adequately predicting outcomes like ADL and I(ADL) disability (Table 5).

**Table 5:** Construct validity (convergent and divergent validity): Pearson correlation coefficients between the scores of TFI domains and self-rated health, PASE, and ADL and IADL, MMSE, GDS, SSC and QoL dimensions

TFI	TFI Ph		TFI Ps		TFI So	
	*r*	*P*	*r*	*P*	*r*	*P*
**Physical Domain**						
ADL	-0. 691	< 0.001	-0.385	< 0.001	-0.268	< 0.001
IADL	-0. 423	< 0.001	-0.235	< 0.001	-0.089	< 0.001
PASE	-0.304	< 0.001	-0.118	0.018	-0.004	0.943
**Psychological Domain**						
MMSE	-0.244	< 0.001	-0.443	< 0.001	0.81	0.719
GDS	0.053	< 0.001	0.300	< 0.001	0.601	< 0.001
**Social Domain**						
Social Support	-0.115	0.022	-0.55	0.272	-0.451	< 0.001
**QoL Dimensions**						
Physical	-0.402	< 0.001	-0.277	< 0.001	-0.251	< 0.001
Psychological	-0.507	< 0.001	-0.363	< 0.001	-0.298	< 0.001
Social	-0.408	< 0.001	-0.307	< 0.001	-0.290	< 0.001
Environment	-0.424	< 0.001	-0.304	< 0.001	-0.307	< 0.001

TFI, Tilburg Frailty Indicator; TFI Ph, Tilburg Frailty Indicator physical domain; TFI Ps, Tilburg Frailty Indicator psychological domain; TFI So, Tilburg Frailty Indicator social domain. QoL: Quality of Life

* One-tailed P value

## Discussion

TFI is a brief-self reported frailty scale, which distinguishes the disability and comorbidity, consists of physical, psychological, and social domains, and takes short time to fill out with short instruction. Additionally, total TFI score was calculated easily for identification and intervention toward frail older people to improve their frailty [49].

The present study aimed to translate and evaluate the psychometric properties of the scale among a sample of community-based older people in Iran. Based on the results of the early studies, TFI is a valid and reliable instrument for assessing frailty [20, 14, 19, 18, and 20]. Test–retest reliability for the total frailty score was at acceptable level (*r* = 0.72), which is not consistent with the results of the previous studies, which were 0.90 among Dutch, 0.91 among Portuguese, 0.87 among German, and 0.88 among Brazilians. Test–retest reliability for physical, psychological, and social domains were also at acceptable level and lower than that to the other related findings [14, 17, 18, and 20], which might be due to the longer interval between two tests in this study (14 days in the present study and 10 days in the other studies).

Convergent validity of the TFI was confirmed by statistically significant kappa coefficient of each TFI item with its related measures. The majority of the TFI items had excellent agreement with related frailty measures. In another study, there was a moderate to high level of agreement between the items of the Tilburg Vulnerability Instrument and the EXTERN scales, except for cognitive performance and the agreement was excellent for living arrangements [50].

The convergent validity of the Persian version of the Tilburg Vulnerability Instrument (TFI) was confirmed by the significant correlation of each dimension with its related scale compared to the unrelated scale. This was well established for the two physical and social dimensions and relatively good for the psychological dimension.

Alternative tools of the TFI psychological domain: MMSE, “Do you have difficulties with your memory?” lacked the strong correlation with their related domain. In addition, some studies indicated that older people’s cognitive impairment had stronger correlation with physical frailty compared to the psychological frailty [14, 18]. Based on the results of other studies, cognitive disorders are related to the development of dependence in the older people [14, 51]. The findings of the present study revealed that the GDS-15 had a stronger correlation with the physical domain and social frailty compared to the psychological domain, which is in line with the following studies on frailty and physical and mental functions. A study among Taiwanese older people [8] found that physical frail participants were more likely to have low SF- 36 scores for the physical and mental domains of the questionnaire, while pre-frail older people indicated low SF-36 scores in only the mental component scale. Accordingly, psychological problems can lead to the low physical functioning. Furthermore, study results of Qi & Li showed that worse social frailty contributes to a significant degree of depression [52].

In the Portuguese community-dwelling older people, six were chosen as a cut-off point for frailty and 54.8% of the participants were frail [20]. The prevalence of frailty was also higher than that in the present study and it can be attributed to the participants’ inclusion criteria of age (≥ 60 years), which was younger than that in the other foreign studies (≥ 65 years). Additionally, in cultures similar to the eastern cultures such as the Iranian’s culture, the older people often live in the family with their children and grandchildren bringing them more mental health and older people Muslims, including Iranians, have a habit of saying “thank God” in the worst situations.

## Conclusion

The Persian version of the scale had good content and face validity and reliability among Iranian older people living in the community, and an appropriate and significant correlation with the physical, psychological, and social dimensions. In addition, P- FTI had a good predictive validity among the older people, which can be used as a screening tool for health and therapeutic interventions. Additionally, it is recommended to assess the validity and reliability of the P-FTI among the older Iranian people and Iranian community dweller who did not receive services from health care centers.

### Limitations and strength

There are some limitations in the present study. First, given that it is a cross-sectional study that precludes the inference about the causal relationship between the frailty and adverse outcomes and cannot assess the predictive validity of the TFI, further studies are needed to use longitudinal cohorts. Second, although the original TFI is a self-administered instrument, a face-to-face interview was adopted, since most of our subjects had a low educational level, which may be time-consuming and affect the accuracy of the results. However, completing the TFI took on average less than five minutes, and all of the Cohen’s kappa coefficients between the TFI items and corresponding alternative frailty measures had statistical significance.

Third, the potential overlap between the items of the TFI and GDS-15 should be considered and the SSRS may induce over-estimation of its predictive validity of depression and social support. Based on the findings, the psychological domain of the TFI overlapped with two items of the GDS-15 (memory and feeling down) and one item of the SSRS (living arrangement). Nevertheless, overestimation is inevitable due to the evidence indicating the intertwining of frailty and depression [53] and therefore, further investigation with extra tools was recommended.

In addition, older people living alone had a higher risk of frailty [54]. Finally, the study was only conducted on the older people receiving services from urban health centers, who may be different from other older people in some aspects such as socioeconomic status, family connections, and support networks.

Conducting the study for the first time in the Iran and applying appropriate sample size are the strength of the present study.

## Data Availability

The present study datasets and analysis sheets are available from corresponding author (E. mail: leilisalehi88@gmail.com) and will be provided through reasonable request.

## Abbreviations

GFI: Groningen Frailty Indicator
TFI: Tilburg frailly index
ADL: Activity Daily Living
IADL: Instrumental activities of daily living
QoL: Quality-of-life
P-PASE: Persian version Physical activity for elderly
BMI: Body Mass Index
TUG test: Timed Up and Go test
MMSE: Mini Mental State Exam
SSRS: The Social Support Rating Scale
APGAR: For adaptability, partnership, Growth, Affection and resolve
WHOQOL: WHO QoL
ICC: Intra-class correlated coefficients
GDS: Geriatric depression scale
